# Capuchin monkeys learn to use information equally well from individual exploration and social demonstration

**DOI:** 10.1007/s10071-022-01654-0

**Published:** 2022-09-05

**Authors:** Donna Kean, Elizabeth Renner, Mark Atkinson, Christine A. Caldwell

**Affiliations:** 1 Psychology, Faculty of Natural Sciences, University of Stirling, Stirling, FK9 4LA UK; 2 Anti-Persoonsmijnen Ontmijnende Product Ontwikkeling (APOPO), Sokoine University of Agriculture, PO Box 3078, Morogoro, Tanzania; 3 Department of Psychology, Durham University, Durham, DH1 3LE UK; 4 School of Management and School of Psychology and Neuroscience, University of St Andrews, St Andrews, KY16 9AJ UK

**Keywords:** Non-human primates, Discrimination learning, Social learning, Individual learning, Touchscreen, Cumulative culture

## Abstract

The limited evidence of complex culture in non-human primates contrasts strikingly with human behaviour. This may be because non-human primates fail to use information acquired socially as effectively as they use information acquired individually. Here, monkeys were trained on a stimulus discrimination task with a win-stay, lose-shift (WSLS) reward structure. In a social learning condition, the experimenter performed an information trial by choosing between the available stimuli; in an individual condition, monkeys made this choice themselves. The monkeys’ subsequent test trials displayed the same stimulus array. They were rewarded for repetition of rewarded (‘win-stay’) and avoidance of unrewarded (‘lose-shift’) information trial selections. Nine monkeys reached our pre-determined performance criterion on the initial two-stimulus stage. Their ability to generalise the WSLS strategy was then evaluated by transfer to a three-stimulus stage. Minimal differences were found in information use between the social and individual conditions on two-stimuli. However, a bias was found towards repetition of the information trial, regardless of information source condition or whether the information trial selection was rewarded. Proficient subjects were found to generalise the strategy to three-stimuli following rewarded information trials, but performed at chance on unrewarded. Again, this was not found to vary by source condition. Overall, results suggest no fundamental barrier to non-human primates’ use of information from a social source. However, the apparent struggle to learn from the *absence* of rewards hints at a difficulty with using information acquired from unsuccessful attempts; this could be linked to the limited evidence for cumulative culture in non-human primates.

**Supplementary Information:**

The online version contains supplementary material available at 10.1007/s10071-022-01654-0.

## Introduction

Human-unique social learning mechanisms have been proposed to explain the complexity of human culture relative to other species. Specifically, humans are argued to rely more heavily on social learning and to engage in higher fidelity copying of behaviour than non-human primates (Boyd and Richerson 1996; Montrey and Shultz 2020; Visalberghi and Fragaszy 1990). This ability to closely match observed behaviour is thought to have allowed the retention of beneficial information over generations and reduced backward slippage in human societies, leading to the emergence of cumulative culture (Lewis and Laland 2012; Tennie et al. 2009). Cumulative culture refers to the development of increasingly complex cultural traits that is made possible by the ability to improve upon the progress of others (Boyd and Richerson 1996; Whiten and van Schaik 2007), examples of which are widespread in humans but relatively scarce in non-humans (Tennie 2009; Dean et al. 2014).

Moreover, cognitive mechanisms have been proposed to have evolved for learning and processing specifically social information in humans (e.g. Cosmides and Tooby 2015; Dean et al. 2014; Henrich 2016; Herrmann et al. 2007; McGuigan et al. 2007; Meltzoff 1988, 1999; Tennie et al. 2016; Tomasello 1999; Whiten 2011). In contrast, although there may be some social influence on learning, non-human primates have been argued to be more restricted in what they can learn socially, depending more heavily on individual learning (e.g. Tennie et al. 2009). Therefore, investigating how non-human primates respond to information from social sources, relative to information acquired individually, represents an important step in establishing whether there is anything different, or special, about social information use in humans. The current study represents an attempt to do just that.

Although the distinctiveness of human culture certainly suggests that social learning is a powerful and important means of acquiring information for humans, the extent to which this process is based upon learning predispositions that are inherently specific to the social domain remains unclear (Osiurak and Reynaud 2019). Adequate comparisons between social and individual learning which could evaluate this question are lacking, despite strong interest in the role of social learning processes in distinctively human culture.

The current study replicated and extended the methods of Renner et al. (2019) who previously investigated this hypothesis using a population of squirrel monkeys. Renner et al. (2019) developed a win-stay, lose-shift (WSLS) task that tested the use of information when stimuli were rewarded or unrewarded (within-subjects), and when the information source was either a social demonstrator or the subject itself (between-subjects). Atkinson et al. (2020) also ran a version of this task with young children to investigate the same hypothesis in relation to child development.

The WSLS structure of the task rewarded subjects for repeating selections that were rewarded (‘win-stay’) and avoiding those that were unrewarded (‘lose-shift’). Each problem involved an information trial where a selection was made between two (Stage A) or three (Stage B) stimuli on a touchscreen computer. This information trial was performed by the experimenter for monkeys in the social condition, and by the subject themselves for those in the individual condition; crucially, identical information was provided across both conditions. That is, the information about the value of the chosen stimulus that was displayed as a result of the information trial was identical regardless of whether the choice was made by the subject (and direct feedback was obtained) or the experimenter (and vicarious feedback was obtained). Test trials immediately followed whereby the subjects selected from the same set of stimuli. If the information trial was rewarded the successful strategy was to repeat the selection made. If it was unrewarded, the successful strategy was to deviate from the information trial selection and choose an alternative. Thus, sometimes the strategically correct response was to copy (‘stay’) and sometimes it was to explore (‘shift’). The variable requirement of either a ‘stay’ or a ‘shift’ response allowed us to investigate information use whilst controlling for effects of prior experience, discussed further below.

A squirrel monkey (*Saimiri sciureus*) population performed poorly on this task, meaning the hypotheses regarding information source could not be fully assessed (Renner et al. 2019). The current study aimed to address this issue by using a population of capuchin monkeys (*Sapajus apella*), where there is some reason to expect greater success, outlined below.

Previous methodologies testing non-human primate social learning have arguably failed to effectively compare social to individual learning. These studies typically involve a social condition where a demonstration provides information about how to successfully retrieve food from a puzzle box, and success is defined by copying this solution (e.g. Davis et al. 2016; Marshall-Pescini and Whiten 2008; Tomasello et al. 1987). This is often compared to an individual exploration condition where no such information is provided but where the subject can freely engage with the apparatus. Evidently, there is no scope for copying in the latter condition, but success requires spontaneously solving the puzzle box during naïve exploration. Thus, there are significant disparities in the information provided between the social and individual learning conditions. In one such tool-use study, Nagell et al. (1993) provided a demonstration of a target behaviour to chimpanzees (*Pan troglodytes*) in a social condition, where a rake-like tool was manipulated to pull food within a reachable distance. An individual control group were given no demonstration but were simply allowed to explore the apparatus. The social group were more successful when subsequently attempting the task, compared with the individual group who were required to spontaneously discover the behaviour. The lack of correspondence between the conditions therefore does not permit conclusions to be drawn regarding the use of information acquired socially compared with that acquired individually.

Several studies have used ghost controls in an effort to establish informational equivalence with a social condition to address this limitation (e.g. Renner et al. 2021; Subiaul et al. 2004). Ghost demonstrations match the object movements or results of a demonstration performed by a social model, but without the perceivable involvement of an actor. For example, Hopper et al. (2007) compared whether chimpanzees’ ability to solve a tool-use task would vary by whether the demonstration was performed by a conspecific or not. Two separate ghost controls involved a demonstration where either the apparatus was automatically triggered, or a tool seemingly operating of its own accord (manipulated using hidden fishing wire) successfully released the food reward. The social demonstration group were significantly more successful than the ghost control group. The authors concluded that the apparatus manipulations must be performed by a social agent for learning to occur. Ghost controls effectively address the informational equivalence limitation by matching both the information provided in each condition and the required response.

However, an issue with such experiments is that a social agent likely draws greater attention to the content of a demonstration than an invisible one (Fawcett et al. 2002). This also likely occurs to differing extents for different species.

More generally, differences in reinforcement history from repeating others’ actions compared with repeating one’s own actions, further impede interpretation of these results. As our goal is to investigate potential learning predispositions, and not the influence of experience, reinforcement history may confound results if only repetition is used to confirm that learning has occurred. Furthermore, the behaviour available for copying is typically not random, but biased towards adaptive variants (Rendell et al. 2011). Experience of the higher likelihood of payoff following repetition of others’ behaviour plausibly creates a generalised positive association with social copying that does not necessarily result from a behavioural predisposition.

This is also particularly problematic for between-species comparisons. Reinforcement history cannot be assumed to be equal between species due to, e.g. differences in species-typical reliance on social learning. Therefore, even when the information content between social and individual learning conditions is matched, defining success by repetition of an experienced behavioural sequence may skew cross-species comparisons. Of course, this is not a design issue with ghost control studies in particular, but with the overarching branch of experiments that compare social to individual learning based on repetition only.

Importantly, the ability to engage in cumulative culture likely requires the ability to discriminatively use information. For example, when observing a conspecific’s problem-solving attempt, an ‘always-copy’ strategy may not be sufficient; instead, copying successful and avoiding unsuccessful attempts may be required for complex culture to evolve (Enquist et al. 2007; Miu et al. 2020).

The current study aimed to minimise these issues by training subjects on the WSLS task i) where identical informational cues were provided across social and individual learning conditions, and ii) that variably required a repeat or a shift response depending on the type of cue provided. As the features of the stimuli vary across trials, each exposure to the task represents a novel problem where associations between particular stimuli and rewards cannot be generalised. This allows multiple task exposures with no carry-over effects and eliminates confounds resulting from differential prior reinforcement histories associated with repetition across the different source conditions. Only through task exposure can subjects learn the WSLS strategy which informs whether a ‘stay’ or ‘shift’ response delivers reinforcement on any given problem.

Possibly partly because of the issues described, there are no clear theoretical predictions in the literature regarding how learning and using information from individual sources compares to social sources for non-human primates. However, non-human primates, thought to be poor social learners relative to humans, might be expected to be at a significant disadvantage when learning from the outcomes of others’ behaviour compared to their own. Firstly, there is limited evidence of overimitation in non-human primates (e.g. Clay and Tennie 2018; Horner and Whiten 2005), and non-human animals more generally have been proposed to lack the “explicit” selective social learning strategies of humans (Heyes 2016). Moreover, examples of culture in non-human primates show few signs of directional modification, i.e. cumulative culture (Tomasello 1999). As previously noted, such evidence has sometimes been interpreted as being congruent with the idea of an adaptation for social learning that evolved late in the hominin lineage. Therefore, it might be predicted that non-human primates would encounter some difficulty, especially relative to humans, in learning the WSLS strategy from a social source.

However, these indicators of apparently poor social learning, relative to individual learning, may be attributable to general cognitive processes, such as the fact that learning from the outcomes of another’s behaviour requires active attention to be paid to the model during the critical moment(s) of the task response. Indeed, monkeys are documented to have poorer executive control of attention than humans (Beran et al. 2016). In contrast, when individually learning, this level of attention is necessarily already enlisted by virtue of direct engagement. As such, absence of an evolved social learning capacity may not be necessary to explain differences in basic social learning propensities in monkeys. If attending to social information is more difficult, or less likely, than to information generated individually, we would also predict slower mastery of the task contingencies for those exposed to social information. However, it would also be expected that any such difference between social and individual information conditions would reduce as subjects became task competent (having learned to attend to cues in the social condition).

The WSLS task enables us to disentangle these influences by examining rates of repetition when learning from each *information source* (social vs individual) and *information type* (rewarded vs unrewarded). The first stage of this experiment (Stage A, 2-stimuli) involved training the subjects on the WSLS strategy, which requires flexible discrimination between cues associated with the presence or absence of rewards. This allowed rates of learning the WSLS rules to be compared between individual and social learning contexts. Any advantage of learning individually at Stage A could be congruent with an inherent difficulty with using socially acquired information, although as noted above, attentional factors could still potentially account for this finding.

Meeting a performance criterion on Stage A signified that the informational cues were reliably attended to and that the strategy was used consistently. We then tested the ability of these proficient monkeys to apply the rules learned during Stage A to Stage B (3-stimuli) with high fidelity, i.e. whether the information was being used by the information receiver in a consistent manner across contexts. ‘High fidelity’ in this context refers to high levels of accuracy in the use of information (e.g. evidence of precise repetition of rewarded selections). Any performance differences shown by proficient monkeys who used the WSLS strategy with high fidelity upon transfer to Stage B (where sufficient attention could be assumed due to reaching criterion performance) would provide strong evidence that the source of information itself influenced the variation. Alternatively, comparable information use between sources, in combination with initial differences in rates of learning, would suggest no fundamental difference in learning in the social domain but might point to other source-related disparities, such as differences in attention.

Only one of the squirrel monkeys achieved proficiency on the WSLS task (Renner et al. 2019), meaning these hypotheses could not be adequately tested. However, a generalised tendency to repeat was found regardless of information source or information type, and it was suggested to potentially represent a pre-existing bias, or a bias that was introduced by the touchscreen training program.

Capuchin monkeys may be a better candidate for acquiring the WSLS strategy, from both individually generated and socially observed cues, as they possess several traits suggestive of capacities for complex culture. For example, there is evidence of traditions in wild groups (Perry 2011; Perry et al. 2003) and capuchins are proficient tool users (Boinski et al. 2000). Capuchins possess allometrically large brains and have a tolerant social system (Perry and Rose 1994), traits thought to be linked to propensities for culture. In addition, capuchins have displayed the ability to copy with relatively high fidelity (Custance et al. 1999; Dindo et al. 2008; Fredman and Whiten 2008; O’Sullivan et al. 2017) even without rewards (Bonnie and de Waal 2007). Of particular relevance to the current paper, young capuchins appear to prefer to observe proficient, over less proficient, nut-cracking models in the wild, suggesting that wild capuchins can learn to discriminate successful behaviour in others to some degree (Ottoni et al. 2005).

To further understand the paradox that many species, including capuchins, demonstrate culture but not cumulative culture, the ability for non-human primates to use social relative to individually acquired information warrants further investigation. Any fundamental difficulties found on the WSLS, particularly if these are restricted to cases where information has been accessed vicariously, may inform our understanding of cognitive barriers to cumulative culture in non-humans.

In Stage A, we expected performance to improve over sessions consistent with learning of the task contingencies. We planned to compare rates of learning between the group trained on cues provided by social demonstration, and the group trained on cues generated by their own responses. We also planned to look for any differences between the two groups in relation to whether the information trial was rewarded or unrewarded.

On Stage B, we compared whether information source influenced generalisation of the WSLS strategy by individuals that had met criterion on the two-stimuli stage.

## Stage A method

This experiment was pre-registered at the Open Science Framework (osf.io/9f26j).

### Subjects and site

Data were collected from two groups of tufted capuchin monkeys (*Sapajus apella*), totalling 35 individuals, housed in separate enclosures at the Living Links to Human Evolution Research Centre at RZSS Edinburgh Zoo. Each group was housed together with a group of squirrel monkeys (*Saimiri sciureus*).

The enclosures were near-identical where each group had access to one indoor (7 × 4.5 x 6 m height) and one outdoor (app. 900m^2^) area, and the research room (during research sessions) where all training and testing was performed. Both indoor and outdoor enclosures were equipped with a variety of enrichment such as grass, trees, shrubbery, large climbing structures and wood chip. Subjects received two main feeds and three to four scatter feeds daily of a mixture of fresh fruit, monkey chow, boiled eggs, meat, insects and vitamin supplements. Water was available ad libitum. See Leonardi et al. (2010) for full details.

Fourteen subjects took part in the minimum number of sessions (see ‘*Procedure**’*) to be included in the analysis (mean age at start of the experiment = 8 years, SD = 4.65; seven females). Subjects were never food or water deprived and all experimental procedures were voluntary and involved no punishment.

### Materials

Subjects were tested in a block of eight Perspex research cubicles arranged in a 2 × 4 matrix. Each cubicle measured 49.5 × 52.1 × 51.4 cm, and cubicles were separated by partitioning slides during testing. The wall of each cubicle facing the computer touchscreen incorporated two holes: one enabled the subject to freely reach through the Perspex to touch all areas of the screen, the other to receive food rewards.

The experimental materials were created using PsychoPy 1.83 (Peirce et al. 2019), and were run on a Microsoft Surface tablet computer connected to an ELO 1939L touchscreen monitor. The stimuli were geometric shapes of varying colours and sizes generated by PsychoPy. The monitor was mounted on a portable trolley for presentation to the subjects. An Apeman camera was used to video record sessions and was attached to the inside of the cubicles in a polycarbonate box. Raisins and sunflower seeds were given as food rewards.

### Design

This study used a mixed factorial design with repeated measures (pre-registered). Information source was a between-subjects variable with roughly equal numbers of subjects assigned to each condition: social (7) and individual (7) (pre-registered). Information type was within-subjects, as all subjects experienced 50% rewarded and 50% unrewarded demonstrations per session (pre-registered). Total number of sessions varied by subject due to individual differences in performance and the voluntary nature of the study. The outcome variables were task success (pre-registered) and repetition of the selection made during the information trial.

### Procedure

Subjects entered cubicles and were voluntarily isolated using sliding partitions. The subjects had previously been trained to signal to end a session and leave the research cubicles at any given point by touching one of the sliding doors. A sunflower seed was given to subjects for isolating in a cubicle, and another on conclusion of the session. Subjects were presented with the ELO touchscreen by pushing the trolley against the front cubicle wall.

#### Training

**Fig. 1:**
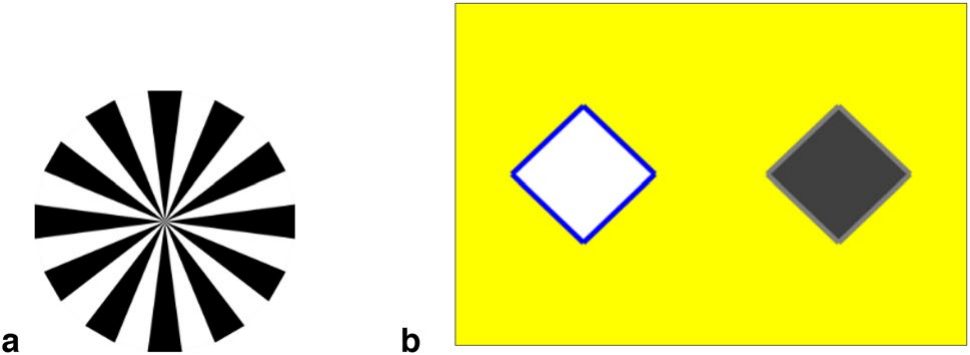
**a** Arbitrary visual reward cue used during training and task, and **b** Stage A example stimuli

As subjects were naïve to touchscreen computers they were initially trained to interact with the screen. This training used shaping and a stimulus fading procedure. Briefly, this involved three consecutive stages where subjects were rewarded with a food item (raisin) and an auditory ‘click’ for (i) touching anywhere on the screen (ii) touching large stimuli on the screen and (iii) touching smaller stimuli (the size used in the task described below). The same arbitrary visual cue (Fig. 1a) as would be used in the experimental task was used to reinforce stages (ii) and (iii) once the stimulus was selected. Each session consisted of ten trials.

Subjects were required to complete 80% of trials per session (8/10) across three consecutive sessions to pass each training stage and progress to the experimental task (pre-registered). Each trial had a time limit of 30 s.

#### Task Procedure

The procedure was identical to Renner et al. (2019) but will be outlined here. There were four problems per session and each problem included one information trial followed by four test trials. For the information trial, the experimenter (social condition) or the subject (individual condition) made a choice between two horizontally aligned stimuli displayed on the screen (Fig. 1b; see example problems in Online Resource 1 video for Stage A, social condition). Stimuli colour and shape (diamond, square, hexagonal) as well as background colour were randomly generated. Colours were within the range known to be discriminable by capuchin monkeys (Waitt and Buchanan-Smith 2006).

Within a session, two problems involved a rewarded and two involved an unrewarded information trial. Their order was randomly allocated by the program whereby the stimulus selected by experimenter or monkey was automatically assigned to the randomly generated information type. In the social condition the experimenter used the function sample() on RStudio (RStudio Team 2014) to randomly order which stimulus would be chosen (left or right). Following a rewarded information trial, the visual reinforcer (Fig. 1a) replaced the stimulus on the screen and the ‘click’ sound was simultaneously produced; subjects in the individual condition also concurrently received a food item (one raisin). Selection of an unrewarded stimulus produced no reinforcers and initiated a timeout of three seconds during which the stimuli disappeared, and the background colour only was displayed.

For each problem, four test trials followed whereby subjects were presented with the same stimuli as the information trial and made one selection per trial. The location of the rewarded stimulus was consistent for each trial within a problem. Only the first test trial was included in the analyses here; the final three test trials (test trials 2–4) were included to scaffold the learning of the predictive relationship between the information trial and test trials, and to be used as part of our performance criteria (see below). Following a rewarded information trial, subjects received all three reward cues (visual, auditory and food) for selecting the same stimulus as the information trial (win-stay; see Online Resource 1 video, first problem, for an example from the social condition) and were not rewarded for choosing an alternative (win-shift). After an unrewarded information trial, subjects were rewarded for choosing the alternative stimulus (lose-shift; see Online Resource 1, second problem) and not rewarded for choosing the same stimulus (lose-stay). If subjects made four consecutive unrewarded selections, there was a brief timeout and a sunflower seed was given to maintain motivation. Subjects had to complete at least one full problem (information trial and all four test trials) for the session to be included in the analysis (pre-registered).

Subjects met criterion on the task when they achieved ≥ 75% correct (i.e. repeating rewarded, and avoiding unrewarded stimuli) on both test trial 1, and the aggregate of test trials 2–4 over three consecutive sessions (pre-registered). If criterion was not met within 30 sessions, testing ceased for that individual (pre-registered). For their data to be included in the analysis, subjects had to take part in a minimum of ten sessions (pre-registered).

There were two opportunities for testing the monkeys per day (am and pm research blocks). Subjects were not tested more than once per block and thus took part in maximum two sessions per day.

All data collection took place from May 2017 to January 2018.

#### Analysis

A logistic generalised linear mixed model (GLMM) (pre-registered) was constructed to test our predictions regarding learning on Stage A.

Task success on test trial 1 was the outcome variable. Responses were recorded as successful if subjects performed a win-stay or lose-shift response and unsuccessful if they performed a win-shift or lose-stay. Information source (social vs individual), information type (rewarded vs unrewarded), session number (as a proxy for experience) and all their interactions were included as fixed effects. Subject ID and location of reward (left or right) were added as random intercepts due to the repeated measures nature of the study and to control for location biases, respectively. Information type was included as a by-subject random slope effect.

All models reported had maximal random effects structures (Barr 2013). Where singular fit or convergence issues were encountered, we adopted a protocol whereby random slopes were initially removed to address this, followed by random intercepts if necessary; however, no such issues were encountered at this stage.

Responses on test trial 1 were recorded as binary for each outcome variable (rewarded [1] vs unrewarded [0]) thus, the models were conducted with a binomial distribution function. To run the models, the binary fixed effects (information source and type) were sum coded so that their levels were -1 and 1. Session number was also centred to account for variation in number of sessions completed by subjects.

A Kaplan–Meier survival analysis and Peto-Peto weighted log-rank test were also performed to determine whether information source (social vs individual) influenced the likelihood of meeting criterion. The survival object included whether the subject met criterion or not as the event, number of sessions as the time variable and information source as a predictor variable. The Peto-Peto modification removes the assumption of proportional hazards and assigns slightly larger weights to earlier events (Karadeniz and Ercan 2017). The latter was appropriate as task experience, which increased with exposure, made the probability of meeting criterion less likely at earlier time points.

Models were created using the glmer function of the *lme4* package (Bates et al. 2014), the survival analysis used the *survival* package, and graphs were created using *ggplot2 and survminer*, all performed on R (R Core Team 2020).

## Stage A results

Nine of the fourteen subjects met our performance criterion, indicating that capuchin monkeys could learn to use the information trial to guide their behaviour. Fig. S1 displays the number of sessions to reach criterion for all subjects, and indicates source condition: individual (the subject itself performed the information trial) or social (the experimenter performed the information trial). Table 1 indicates the proportion of success on test trial 1 for all subjects (un-averaged).

**Table 1:** Proportion of WSLS strategy success for all subjects across all trials, un-averaged. Chance level is 0.50

	WSLS success
Information source	
Individual	0.56
Social	0.59
Information type	
Rewarded	0.74
Unrewarded	0.39

### WSLS success GLMM

**Fig. 2:**
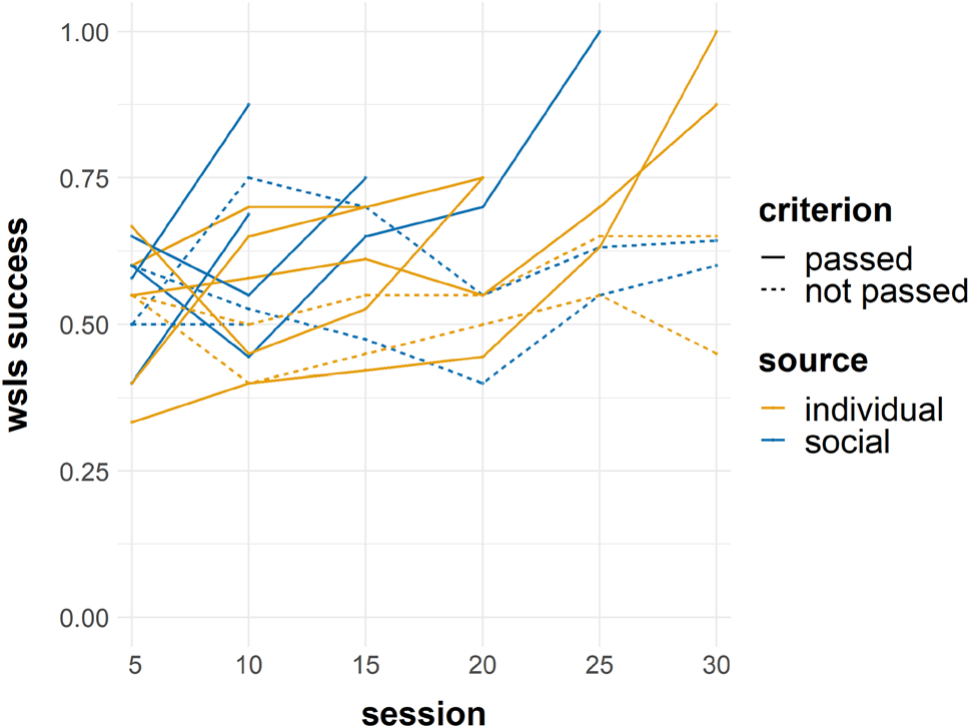
WSLS success over sessions separated by subject, whether criterion was met or not, and information source. Sessions were grouped into blocks of five. NB: includes the three sessions where criterion was met

**Fig. 3:**
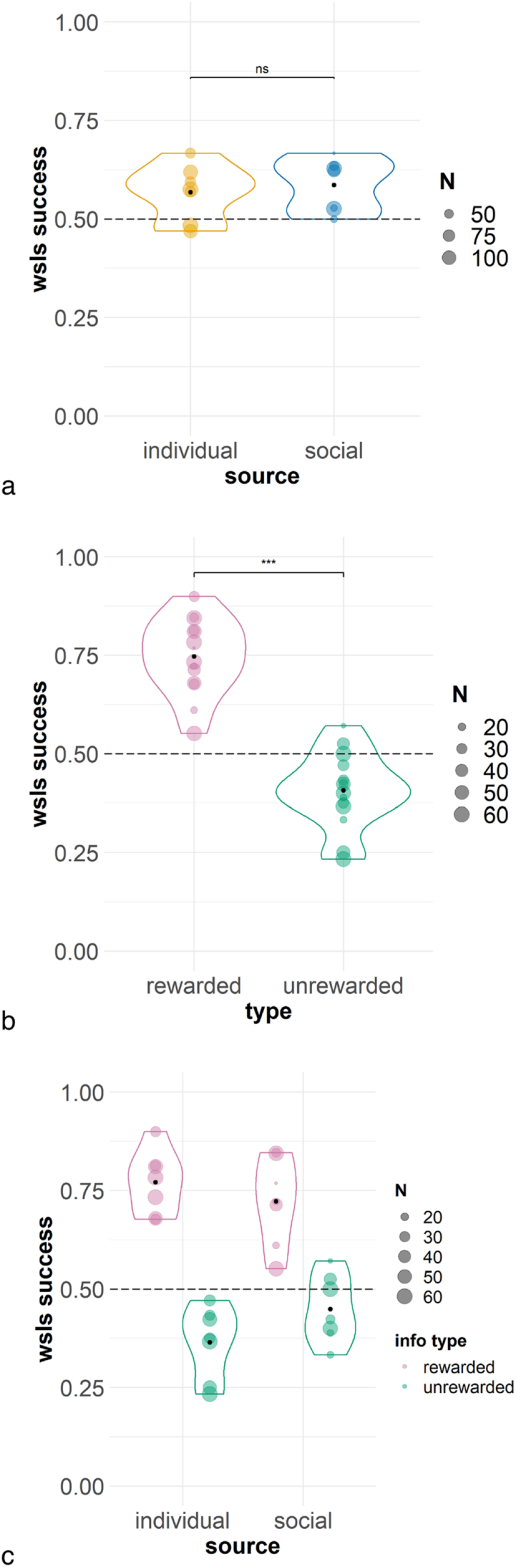
WSLS success on Stage A for all subjects by information source (top), information type (middle), and the interaction between information source and type (bottom). Dashed line indicates chance and the black point indicates the mean. ‘*N*’ indicates the number of data points (trials) for each individual subject

The ‘success’ GLMM was significantly better than its null equivalent (*X*^2^(7) = 38.57, *p* < 0.001) and predicted the data well under AIC (1360 vs 1384.6); however, by BIC there was a suggestion that the model could be over-parameterised (1415 vs 1404.5). Overall, increased success was found with exposure to the task (session number; *b* = 0.02, SE = 0.009, *z* = 2.22, *p* = 0.03) indicating that performance improved with experience, which appears to be driven by subjects that met criterion (Fig. 2). Significantly greater success on rewarded compared to unrewarded problems (*b* = 0.76, SE = 0.09, *z* = 8.77, *p* < 0.001) (Fig. 3) indicates that subjects performed better when a win-stay response was required, compared with lose-shift. Figure. S2 displays subjects that met criterion only and suggests strong performance on rewarded problems from the outset, whilst responses to unrewarded problems were initially poorer but generally appeared to improve by the conclusion of this stage.

Information source was not a significant predictor (*p* = 0.55) (Fig. 3), suggesting no differential effect depending on whether the information was generated by the experimenter or the monkey itself. However, it is also possible that limited power may have hindered detection of an effect. Finally, the interaction between information source and type fell just short of our criterion for significance (*p* = 0.056). The remaining interactions were not significant (*p* ≥ 0.31) (Fig. 3). Thus, the source of information also did not appear to influence learning on this task or response to each information type.

#### Survival analysis

Finally, a Kaplan–Meier survival analysis curve was constructed as an additional test of the contribution of information source to the learning process; this perspective assessed whether the social or individual condition was predictive of number of sessions to reach criterion (Fig. S3, Online Resource 2). This analysis accounted for the possibly confounding issue that subjects who met criterion at an early stage necessarily contributed less data to the GLMMs described above than those who required more sessions.

Nevertheless, a log-rank test found that information source had no effect on likelihood of reaching criterion; the ‘risk’ of meeting criterion in the social group was not significantly different to the individual group (χ^2^(1) = 0.6, *p* = 0.4).

#### Repeats

To ascertain whether the subjects simply performed the same response (repeat the information trial) regardless of the outcome of the information trial (Table 2), we constructed a second model that was identical to the first except that repetition was used at the outcome variable: if the choice made during the information trial was repeated on test trial 1, we classified this as ‘repetition’, and choosing an alternative was a ‘shift’ response. Session number was also not included in this model.

**Table 2:** Proportion of repetition for all subjects across all trials, un-averaged. Chance level is 0.50

	Repetition
Information source	
Individual	0.71
Social	0.63
Information type	
Rewarded	0.74
Unrewarded	0.61

**Fig. 4:**
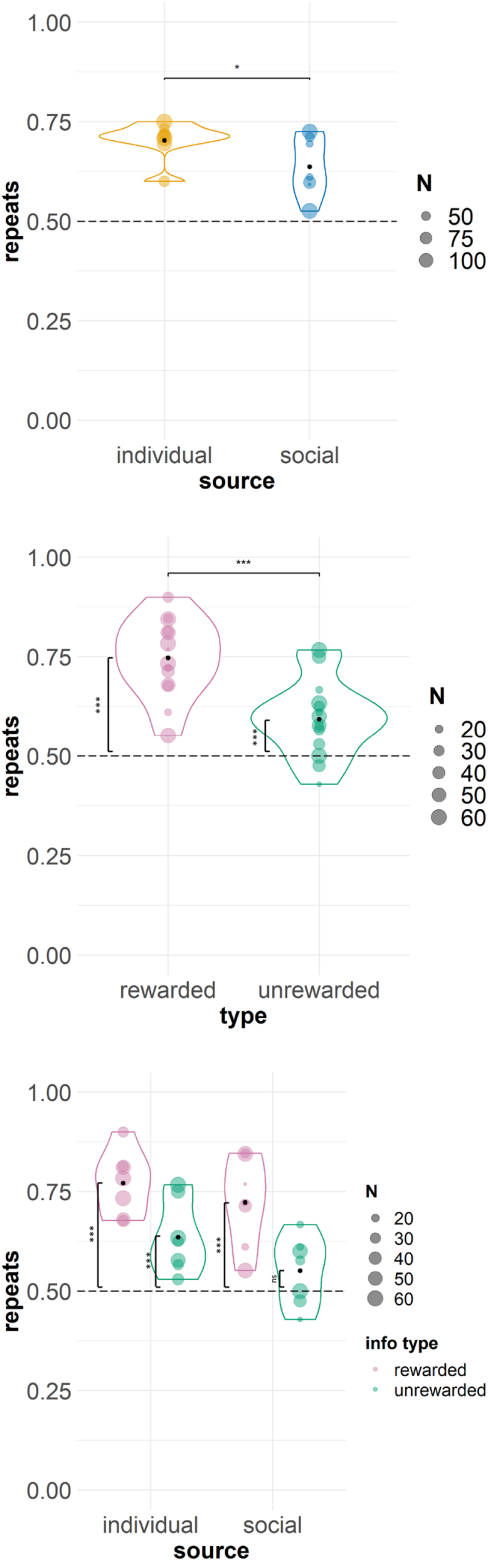
Repeats on Stage A for all subjects by information source (top), information type (middle), and the interaction between source and type (bottom). Horizontal significance brackets indicate group differences. Exact binomial tests found that monkeys repeated above chance on both rewarded and unrewarded problems overall (both *p* < 0.001; middle panel, vertical significance brackets indicate difference to chance); when also separated by source, monkeys repeated significantly above chance (bottom panel) on all (*p* < 0.001) except unrewarded problems in the social condition (*p* = 0.19). NB: For the rewarded information type, successful and repeat responses are iso-directional; for the unrewarded information type, successful and repeat responses are in opposite directions. Dashed line indicates chance and the black point indicates the mean. ‘N’ indicates the number of data points (trials) for each individual subject

The repetition model was significantly different to the null model (*X*^2^(3) = 16.33, *p* < 0.001), and again it was a better fit of the data by AIC (1360.1 vs 1370.5) but not BIC (1395.1 vs 1390.5). Information source was a significant predictor, such that the individual condition (*b* = 0.17, SE = 0.08, z = 2.05, *p* = 0.04) (Fig. 4) was associated with increased repeats. Thus, subjects in the individual condition repeated information trial selections more than the subjects in the social condition. Consistent with the reinforcement contingencies of the task, the rewarded information type was associated with significantly more repeats than unrewarded (*b* = 0.35, SE = 0.08, z = 4.16, *p* < 0.001) (Fig. 4). The interaction between source and information type was not significant (*p* = 0.60) (Fig. 4).

## Stage A discussion

Over half of the subjects met criterion, indicating that they could strategically use the cues provided in the information trial to some degree, contrasting with the minimal progression of squirrel monkeys on the same task (Renner et al. 2019). However, several subjects did not appear to show improvement, even across the maximum thirty sessions, implying limited awareness of the predictive relationship between the information trial and the test trial.

Learning of the WSLS strategy was not found to significantly vary by whether information was provided through social demonstration or individual experience, despite robust analysis to ensure a potential effect had not been missed. This suggests that because the value of information provided was strictly matched, and repetition was not the only rewarded response, learning to use social information occurred just as readily as learning to use individually acquired information. This finding implies the learning cues were equally potent in this context.

Subjects were found to perform the win-stay response more than the lose-shift; at first glance, this may suggest that the subjects were more proficient at learning the win-stay rule. However, this is unlikely considering that above chance levels of repetition were found i) across both information trial types, and ii) following minimal exposure to the task. These points instead indicate that repeat was simply the dominant response overall, irrespective of the learning that occurred as a result of task experience.

Thus, an explanation that better fits our pattern of results is that the monkeys had a pre-existing generalised bias to repeat, largely regardless of whether this was repetition of their own behaviour or the demonstrator’s. This corresponds to the tendency to repeat found by Renner et al. (2019) with squirrel monkeys. The appearance of the reward cue, already reinforced in training sessions, may have broadly heightened interest in the associated stimulus, driving the slightly higher rates of repetition in the rewarded condition. As such, these findings are possible without invoking a strategic understanding of the task contingencies, or indeed suggesting that the monkeys treated the information trial as information at all. Also similarly to Renner et al.'s (2019) 3D version of the task (experiment 2), we found slightly higher rates of repetition in the individual learning condition. Simple behavioural inertia (individual condition) and stimulus enhancement (social condition) likely underlie this repetition bias (Renner et al. 2019).

However, it is plausible that subjects that did meet criterion (henceforth ‘criterion subjects’) entered the study with this spontaneous bias to repeat but began to treat the information trial as ‘information’ that they used with an element of cognitive control as the WSLS strategy was acquired. In other words, as the predictive relationship between information trial and test trial was repeatedly experienced, criterion subjects may have begun to copy rewarded and inhibit repetition of unrewarded selections, resulting in increased success consistent with a rule being learned (Fig. S2).

Importantly, the low levels of learning during most sessions included in the Stage A analysis (i.e. all of the non-criterion monkeys’ sessions and the early sessions of the criterion subjects) may have obscured a potential effect of information source, which was predicted to occur when learning was actually taking place. As learning presumably occurred mainly in the sessions immediately before criterion was met, any influence of information source may be expected only during those sessions.

**Fig. 5:**
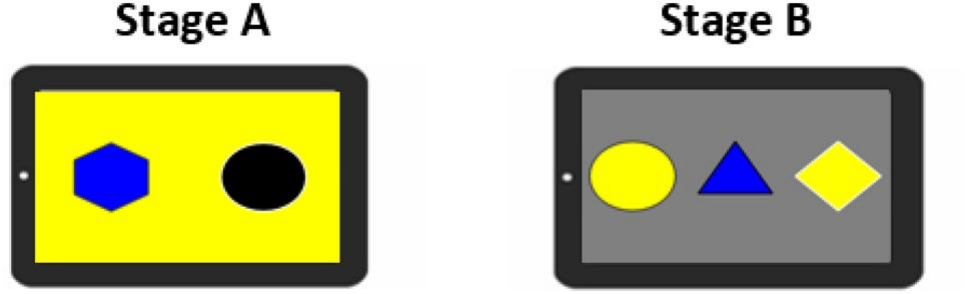
Example stimuli from each stage. One selection was made, and one stimulus rewarded at both stages

To deepen our understanding of the influence of information source on strategic information use by criterion subjects, we presented them with a three-stimulus version of the task in a second stage (B); again, only one stimulus was rewarded (Fig. 5). Increasing the number of distractor stimuli reduced the likelihood of repetition by chance; the ability to generalise the WSLS strategy was then evaluated by comparing performance from Stages A and B.

Although we found minimal effects of source in Stage A, we were interested in whether the source of information may affect the fidelity of information use on transferral to a new context. This may be key to understanding the apparent lack of evidence for cumulative culture: perhaps animals only have the capacity in very specific contexts, and information gained may not be applied in other contexts. If information were used with high fidelity, we might expect for example that rewarded stimuli would be repeated at similar rates regardless of the number of distractor stimuli. Such responding would suggest that the WSLS rules had been learned. However, if rates of repetition of rewarded stimuli were lower in Stage B relative to Stage A, this would be more consistent with low-fidelity use of information. Essentially, the question is, have the monkeys learned *to repeat* (or to avoid) one particular selection through their exposure to its reward value (in which case we would expect to see this applied with high precision)? Or alternatively, does the information trial simply induce a bias towards a particular selection such that rates of repetition will be strongly affected by the baseline probability of making that selection relative to alternatives?

Because most sessions included in Stage A occurred before any of the WSLS strategy was acquired, i.e. before criterion had been met, we considered a subset of the criterion monkeys’ data for the transfer analysis (see ‘analysis’ section below) to mitigate the potentially ‘noisy’/diluting influence of pre-acquisition sessions. This analysis may constitute a more valid test of the influence of information source by considering only the most proficient monkeys who had presumably engaged in at least some learning to attend to the task cues. Although this translates to a small dataset, we believe these are worthwhile analyses due to their ability to speak to the fidelity of information use, which has taken a central role in discussions of comparative cumulative culture.

As the information provided on unrewarded trials did not appear to have been used with proficiency on Stage A, any improvement in response to unrewarded information was also of interest. Because the successful response to unrewarded trials is incongruent with the monkeys’ predisposition to repeat found on Stage A, the bias may take significant effort to overcome.

In the following section we examined whether information source affected generalisation of the WSLS strategy on transfer from two- to three-stimulus arrays. Any consistent differences found between the source conditions here would suggest that processing of information is fundamentally affected by the origin of that information.

## Transfer method

Stage B was identical to Stage A except for some key details outlined below. Information source condition assignment remained constant across both stages.

### Subjects

Nine monkeys reached our pre-determined performance criterion on the two-stimulus task (Fig. S1); seven out of these nine (five from the individual and two from the social condition; henceforth ‘criterion subjects’) were transferred to Stage B to assess their ability to generalise the WSLS strategy. For the two of the original nine subjects that had met criterion but were not transferred, there was a gap of more than two weeks between reaching criterion for Stage A and the beginning of Stage B. Thus, they were required to meet criterion again over two sessions on Stage A to be transferred but did not achieve this. Since the intention was to test only individuals who had achieved proficiency in Stage A so that we could determine how this learning was generalised, these individuals were not included in the Stage B testing.

### Design

Stage (A and B) was added as a categorical variable.

### Procedure

The key task alteration was that the number of stimuli available to choose from increased from two to three. As in Stage A, one stimulus was rewarded (the other two were unrewarded), and two information trials per session were rewarded and two were unrewarded. However, one problem per session during Stage B required subjects to choose an alternative stimulus twice to locate the rewarded stimulus (see Online Resource 3 video for an example from the individual condition); use of the correct strategy on test trial 1 (i.e. shift) regardless of finding the target was recorded as a correct trial. As test trial 1 only was included in the analysis, the outcome of the subsequent test trials did not affect the results.

#### Analysis

A logistic GLMM was performed on the Stage B data only to test the influence of information source and type on the 3-stimuli stage. As with the Stage A GLMMs, data were included for all criterion monkeys up to their thirtieth session for Stage B. The model included repeats on test trial 1 as the outcome variable, and information source, information type and their interaction as fixed effects. Subject ID was included as a random intercept and information type was included as a random slope. All fixed effects were sum-coded by the same processes as in the Stage A GLMMs. Information type was dropped as a random effect due to non-convergence.

The last three sessions before criterion was met on Stage A and the first three sessions of Stage B were included in the following analyses (binomials and chi-squares). This allowed comparison between conditions once subjects had reached a suitable level of competence with the task. The three sessions where subjects met criterion were not included in any analysis as this would necessarily include sessions where success of 75% or above was achieved. However, these are included in the figures for illustrative purposes only.

Four exact binomial tests were performed to test whether proportion of repeats on test trial 1 was significantly different to chance for each combination of levels: Stage A, rewarded; Stage B, rewarded; Stage A, unrewarded; and Stage B, unrewarded.

Two chi-square tests (one for each information type) were also performed to determine any statistical differences between proportions of repetition across stage (A vs B).

The binomial tests were conducted using the binom.test function and the chi-squares using chisq.test, all on R (R Core Team 2020).

## Transfer results

### Repeats GLMM

**Fig. 6:**
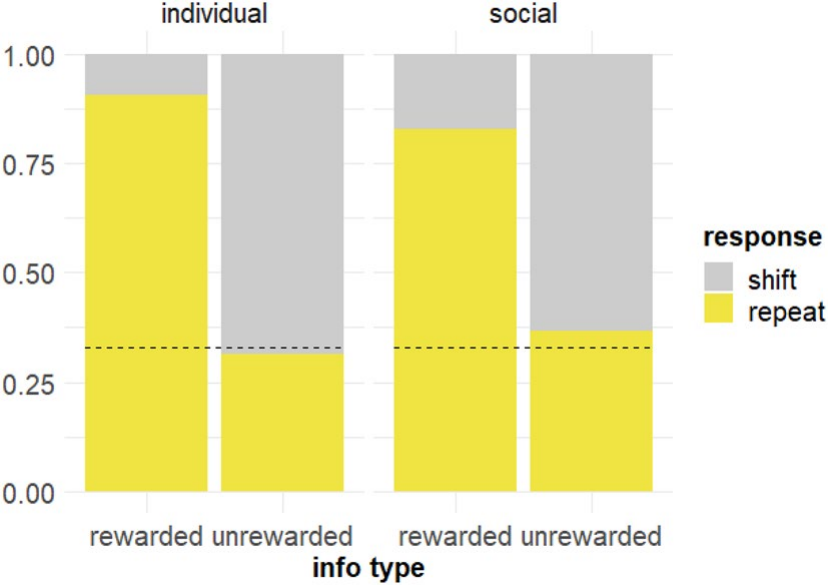
Repeats on all sessions of Stage B broken down by information type and by information source. The optimal response for rewarded trials was to repeat, and for unrewarded was to shift. Dashed line indicates chance

The GLMM was significantly more explanatory than the null model (*X*^2^(3) = 150.25, *p* < 0.001). The only significant effect was of information type (*b* = 1.30, SE = 0.12, *z* = 10.62, *p* < 0.001) (Fig. 6), as there were significantly more repeats following rewarded compared to unrewarded trials, in-line with the correct strategy.

Information source (*p* = 0.47) (Fig. 6) did not have a significant effect on repetition, and the interaction between type and source was non-significant, although close to our alpha criterion (*p* = 0.051).

### Binomial and chi-square tests

All results in the remainder of this section will be considered relative to the associated chance of repetition (Stage A = 0.50; Stage B = 0.33). Table 3 displays the proportion of repetition at each stage.^1^ Here, above chance reflects good performance on rewarded problems, but poor performance on unrewarded problems (and vice versa for below chance).

**Table 3:** Proportion of use of repeats, split by information source. The three sessions prior to meeting criterion on Stage A and the first three sessions of Stage B are reported

	Rewarded	Unrewarded
Repeats		
Stage A (pre-crit)		
Social	0.67	0.58
Individual	0.83	0.72
Stage B		
Social	0.92	0.42
Individual	0.77	0.34

Rewarded problems.

**Fig. 7:**
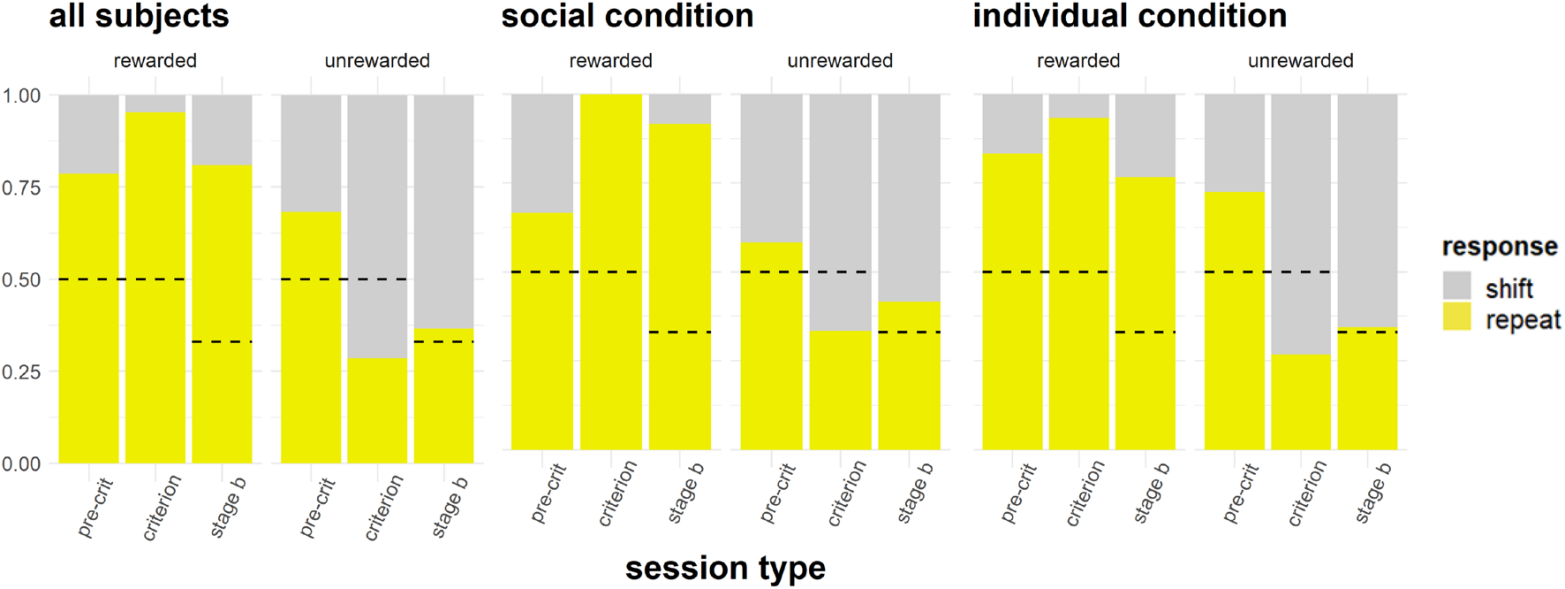
Response (shift or repeat) to rewarded and unrewarded information trials by all criterion subjects (left) and separated by information source (middle and right). The last three sessions of Stage A before criterion was met (“pre-crit”), three sessions where criterion was met on Stage A (“criterion”, included for illustration only) and first three sessions of Stage B (“Stage B”) are shown. The optimal response for rewarded trials was to repeat (above-chance performance), and for unrewarded was to shift (below-chance performance). Dashed line indicates chance (Stage *A* = 0.50; Stage *B* = 0.33)

Two exact binomial tests found that the proportions of rewarded information trials repeated during Stage A (0.79; *p* < 0.001) and Stage B (0.81; *p* < 0.001) were significantly greater than the expected chance proportions of 0.50 and 0.33, respectively (Fig. 7).

Furthermore, a chi-square test of independence confirmed that there was also no statistical difference between proportion of repeats across the stages (*χ*^2^(1) = 0.07, *p* = 0.79). Thus, the drop in chance of repetition from introducing more distractors did not result in any decrease in rates of repetition, suggesting the information was used with high fidelity.

#### Unrewarded problems

The proportion of unrewarded information trial selections repeated during Stage A (0.68; *p* = 0.03) was also greater than by chance; however, by Stage B (0.37; *p* = 0.74) subjects were not significantly different to chance (Fig. 7). A chi-square test of independence found a difference in the proportions of repeats across Stage A and B, which is what would be expected given the change in stimulus number (*χ*^2^(1) = 8.26, *p* = 0.004).

These results suggest that on acquiring the WSLS strategy on Stage A (i.e. meeting criterion) and progressing to Stage B, capuchins’ repetition of unrewarded demonstrations went from greater than chance (poorer performance) to chance levels.

## Transfer discussion

We found no evidence that there was any fundamental difference in the fidelity with which information was used between the individual and social learning conditions when the number of distractor stimuli was increased, considering only subjects of higher task proficiency. This is similar to the results of Renner et al. (2021) who found little influence of source condition on proficient monkeys in a within-subjects variation of the current task (carried out after the current study).

Our results do suggest that our criterion subjects had learned the win-stay rule, as their ability to successfully respond to the reward cue (by repeating) was found to be robust across contexts, despite the change in task structure on Stage B of this study. In addition to simply being significantly above chance, a similar level of repetition was maintained on progression to Stage B, implying minimal variation in rates of copying was generated by the introduction of another distractor. As such, this species does appear capable of learning and generalising a simple rule-based strategy.

As subjects did not master the lose-shift response, we cannot fully test the generalisation hypothesis in this condition. Learning the required response for unrewarded information trials may have been more difficult for this species as it involves inhibition of a pre-existing tendency to repeat (as previously discussed). This contrasts with rewarded problems where this tendency was congruent with the optimum strategy, which was therefore frequently positively reinforced. Although the subjects did not approach proficiency on unrewarded problems, there may have been a trend towards decreased repetition (from significantly above chance to non-significantly different from chance) on transfer to Stage B. Of course, this is somewhat conjectural given the small quantity of data included in this analysis. However, potentially, as our subjects became selectively more attentive to stimuli associated with the reward cue, they became less attracted to unrewarded stimuli which were not linked with this cue. The chance level performance on unrewarded problems in Stage B may indicate that unrewarded information trial selections stimulated no particular interest in any of the available stimuli, whereby the unrewarded cue was still not treated as ‘information’. This relatively neutral approach may be a precursor to actively avoiding unrewarded stimuli, and thus engaging in the optimal lose-shift strategy. This may require increased exposure to the task.

Overall, we found that information about the location of rewards was used with reliability in a fresh context, although the poor performance on unrewarded problems precludes any conclusion regarding the fidelity of use of this type of information. Notably, generalisation of the WSLS strategy was found to have no link to whether the information was provided socially or from individual exploration.

## General discussion

Our aim was to train one group of monkeys to learn a WSLS strategy using cues from a social source and another group with cues from individual exploration. The WSLS task is a robust method for comparing these conditions owing to its capacity to control for variation in information delivery between sources and in reinforcement history.

Comparisons between monkeys in the social and individual conditions found no evidence that either the rewarded or unrewarded cues were learned or used differently across the two groups, nor that information was used with higher fidelity in either group. This suggests that in principle, the study species learned to use social information as effectively as they learned to use information based on feedback from their own activity.

A study testing children recruited in Scotland and China on a homologous version of our task also found that information source had no effect on 2- to 5-year-old children’s performance (Atkinson et al. 2020). This similarity between the human and monkey populations implies that, fundamentally, learning from social sources may not be driven by a specialised cognitive adaptation (even in humans) but could be based on general-purpose learning mechanisms (Behrens et al. 2008; Heyes 2012; Osiurak and Reynaud 2019). Potentially, at least some of the significant differences found between humans and other animals in typical social learning studies could be attributed to variation in general learning capacities. Of course, there are likely facets of human cognition that are specific to the social domain that do play an important role in cumulative cultural evolution. These may be necessary for more challenging tasks than the current one, e.g. those requiring the application of information based on theory of mind or meta-cognitive reasoning (Dunstone and Caldwell 2018; Heyes 2016).

Our results may contrast with theories which implicate a fundamental difference in the treatment of information gained through direct experience or from social sources, both within and between species. However, other lines of research indicate that our results are not surprising, such as neuroimaging studies reporting that personally executed vs. observed actions activate the same neural pathways (Bonini and Ferrari 2011). More specifically, this has also been observed when personally experiencing vs. observing negative feedback in humans (Shane et al. 2008; Yu and Zhou 2006).

The present study did find that the capuchin monkeys struggled to grasp the significance of the task cues, particularly on unrewarded trials. A bias towards repetition of behaviour was found, driving high rates of repeat responses to both ‘win’ and ‘lose’ problems. It may be that primates have a general difficulty with selective learning, involving sub-optimal adjustment of responses relative to the available information regardless of the information’s source.

Atkinson et al. (2020) found that children successfully adopted both the win-stay and the lose-shift rules, using both types of information with high fidelity. However, the children also displayed a striking tendency to explore novel locations (see also Blanco and Sloutsky 2020), performing far better following *unrewarded* information trials, compared with rewarded ones. These results contrast strongly with the bias towards repetition found in the capuchins. Furthermore, the children’s performance implied a solid grasp of the task contingencies and flexible information use, whereas the monkeys’ results suggest only limited recognition of the significance of the cues to the reward location.

Despite entering the task with a repetition bias, our criterion subjects appear to have developed some selectivity in their use of the information with task exposure. The generalisation of the win-stay rule indicates that this type of information was used with precision. The trend towards inhibition of lose-stay responses also appears consistent with this interpretation. Developing an understanding that the reward cue resulted in reinforcement for repetition possibly cultivated a strategy such as ‘repeat-when-reward-cue-appears’, which may have rendered the subjects less susceptible to the repetition bias when faced with an unrewarded information trial, and therefore less drawn to the stimulus that was selected.

The shift from reflexive responding to a more strategic approach was likely easier for the win-stay rule, where the repeat bias was congruent with, and positively reinforced by, the task structure. Learning what *not* to do may have been more difficult because the unrewarded cue necessitated a response that conflicted with the demonstration (Brown and Braver 2005). This may be surprising considering repetition after an unrewarded trial systematically produced a timeout and many unrewarded trials were experienced by each subject across this experiment. This may also have been partially due to subjects’ reinforcement history, e.g. a pre-existing more powerful association with repeating rewarded behaviour due to the immediate gain, relative to active avoidance of unrewarded behaviour, which is less likely to result in instant gratification. However, the small number of trials included in the analysis might also have contributed to this finding.

A limitation of the current study is the sacrifice of ecological validity in favour of greater experimental control. However, we contend that the abstract nature of our task was necessary to robustly investigate information use when *only* the information source had varied, which was our overarching research question.

The implementation of the WSLS task in the current study involved a degree of memory load for the information trial to be used to guide test trial selections. It was important for attention to be directed both to which stimulus was selected and to whether it revealed the reward cue. It is plausible that this cognitive load made the WSLS strategy difficult for the non-criterion monkeys to learn, regardless of information source, and for mastery of the lose-shift rule overall (Wilks et al. 2021). Indeed, working memory capacity has been linked to the emergence of sophisticated culture in humans (Guida 2020; Wynn and Coolidge 2007). It has also been documented that non-human primates are more prone to bottom-up, habitual responding than humans who exercise greater executive control of attention (Beran et al. 2016).

Overall, we found an interesting pattern of results that may have implications for understanding why animals display behaviour consistent with cultural learning but little evidence of cumulative culture. The ability to overcome natural biases and approach problems strategically may amount to a significant barrier at the group level where cultural accumulation occurs. Particularly, learning to disregard actions that were unrewarded may present cognitive challenges that are too difficult to overcome for non-human primates; for example, storage of negative-feedback information in order to avoid repeating unsuccessful behaviour in future. In comparison to humans, explicitly strategic, high-fidelity use of information may be less available to non-human primates, which may significantly contribute to the scarcity of cultural accumulation.

The body of results presented here and by Atkinson et al. (2020) and Renner et al. (2021) suggests that children and capuchin monkeys are, at least in their performance, indifferent to whether information is derived through social or individual learning when the conditions for learning are controlled. Therefore, it is likely that general-purpose learning abilities may at least partially account for differences found between humans and other primates on tests of cultural learning. Future experiments should ensure an adequately matched asocial learning control to test the nature of social information use more comprehensively. However, barriers to general selective learning may constrain the ability for non-human species to repeat useful behavioural variants and intentionally disregard less effective alternatives. Our understanding of why human culture significantly differs from that of other species at a basic level would benefit from a finer grained investigation of the selectivity of learning.

## Supplementary Information

Below is the link to the electronic supplementary material.

Supplementary file1 (TS 44049 KB)

Supplementary file2 (DOCX 174 KB)

Supplementary file3 (MP4 16438 KB)

## Data Availability

The full datasets generated and analysed by the current study are available in the Open Science Framework (OSF) repository, https://osf.io/9f26j/.

## Footnotes

1 The breakdown in the number of trials included in each analysis in this section are as follows: three sessions per stage (A and B), two problems per information type (rewarded vs unrewarded), for seven monkeys (3 * 2 * 7) = 42 trials per information type, per stage. One monkey (Flojo) from the individual group did not complete one trial per stage, meaning there were 41 trials per stage for the individual condition.

## Code availability

The R script including all analyses performed is available at the OSF repository, https://osf.io/9f26j/.
