# Supplementary material for: Capuchin monkeys learn to use information equally well from individual exploration and social demonstration
Source: Anim Cogn. 2022 Sep 5;26(2):435–50. doi: 10.1007/s10071-022-01654-0 (PMC9950169; doi:10.1007/s10071-022-01654-0)
Supplement: Supplementary file 2 — Supplementary file2 (DOCX 174 KB) [file 10071_2022_1654_MOESM2_ESM.docx]

# Online Resource 2


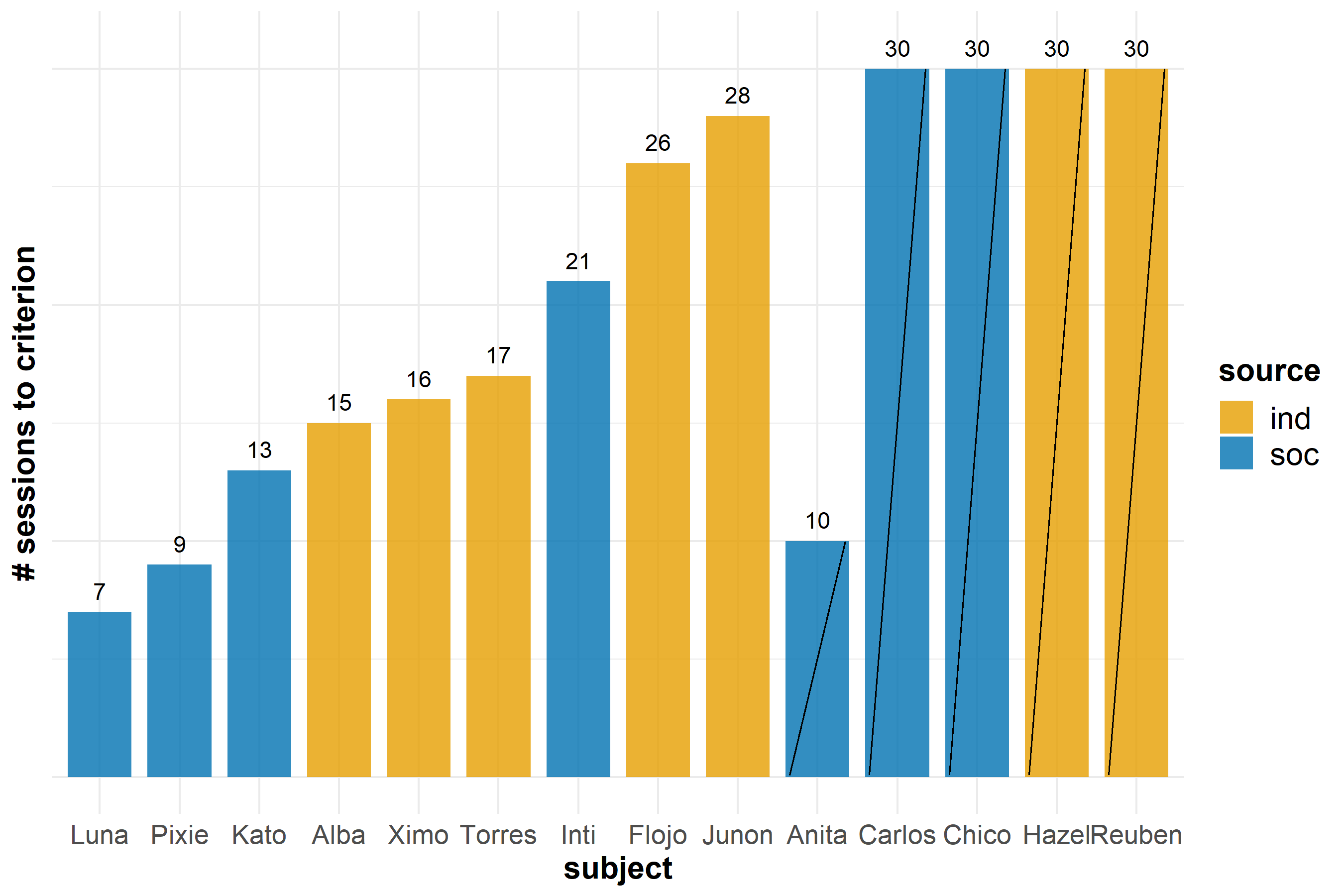


**Fig. S1** Number of sessions to reach criterion and condition of information source received. Diagonal line through indicates subjects who did not meet criterion.


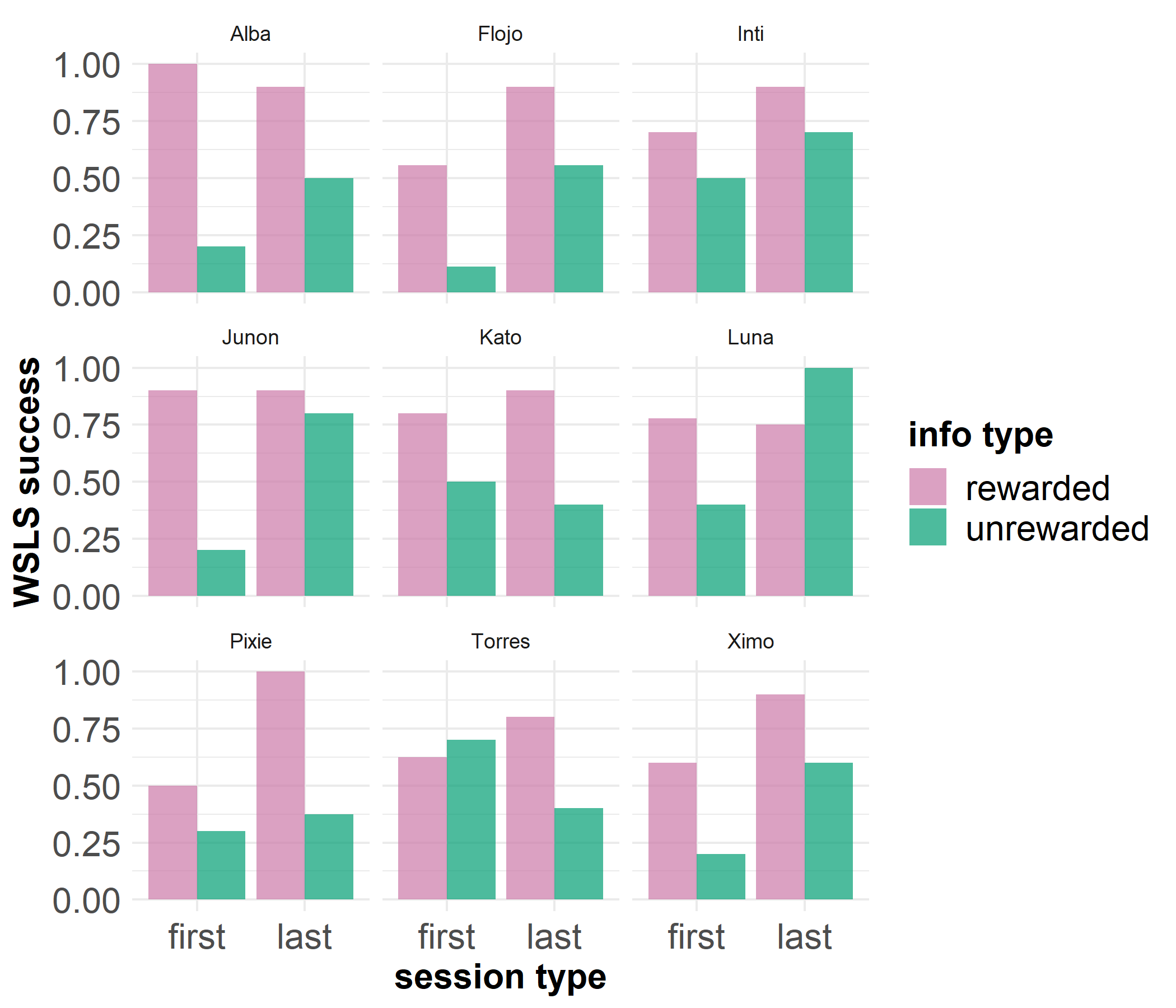


**Fig. S2** Use of WSLS strategy by subjects that met criterion separated by information type. The first and last five sessions only are included as a broad overview to each subject’s progress on Stage A. This includes the three sessions where criterion was met.


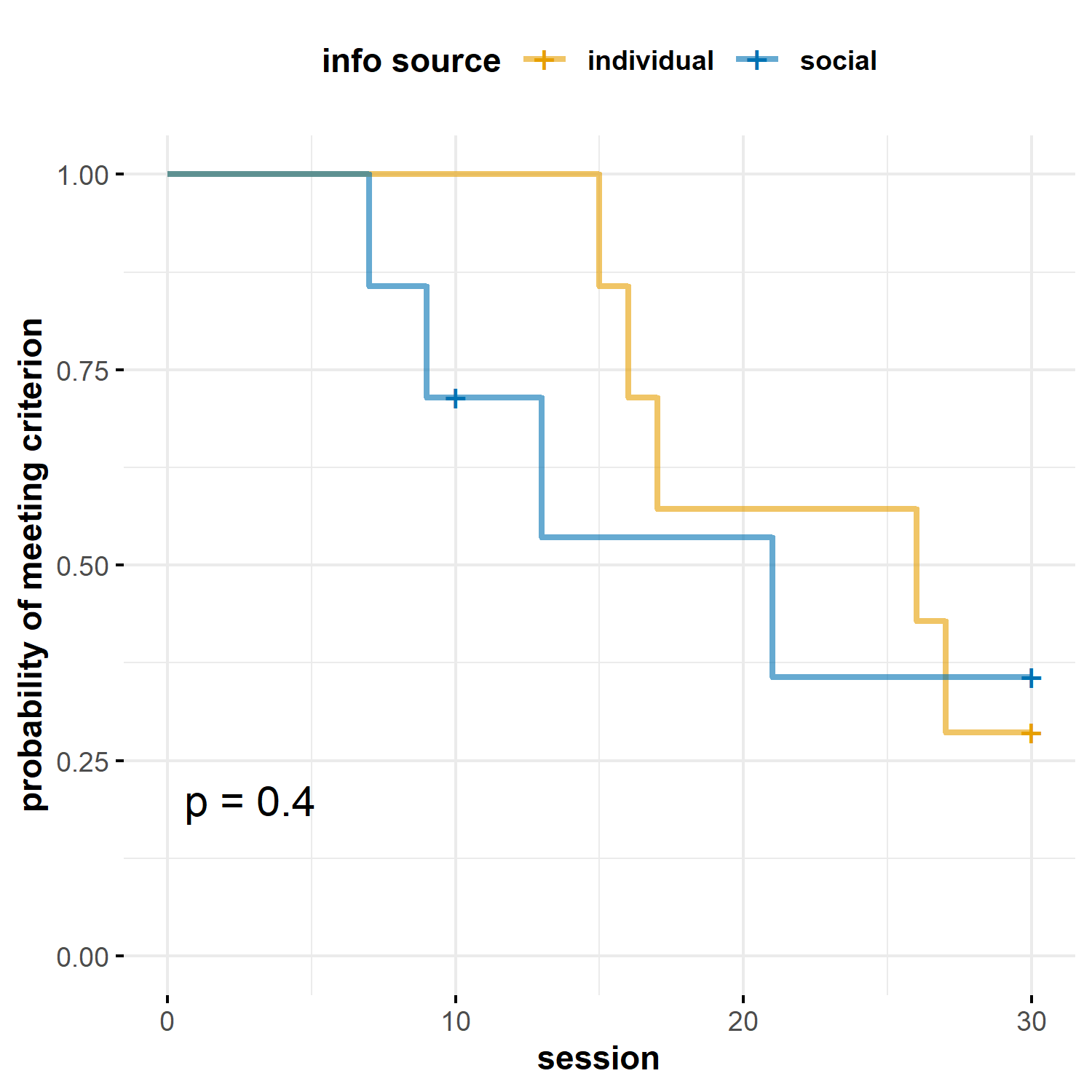


***Fig. S3*** *Kaplan-Meier curves displaying the probability of meeting criterion over sessions for each information source.*
